# Reply to Paul R.B. Kozowyk: Interpreting the complexity of archaeological adhesives may lead to misconceptions about early humans

**DOI:** 10.1073/pnas.2300325120

**Published:** 2023-02-07

**Authors:** Patrick Schmidt, Tabea J. Koch, Claudio Tennie

**Affiliations:** ^a^Department of Early Prehistory and Quaternary Ecology, Eberhard Karls University of Tübingen, 72070 Tübingen, Germany; ^b^Applied Mineralogy, Department of Geosciences, Eberhard Karls University of Tübingen, 72070 Tübingen, Germany

Kozowyk (1) proposes an alternative interpretation of a study on Neanderthal birch tar making (2). He compares findings from 2019 that birch tar can be made with a simple aboveground method—the then unknown condensation method—with more recent results on *Podocarpus* tar making in the African Middle Stone Age (MSA) (3). He argues that if *Podocarpus* tar making can yield information about ancient cognition, birch tar making can, too. We agree with this. However, the rationale behind Kozowyk’s (1) proposition is based on two misconceptions, highlighting that complexity interpretations remain problematic.

1) The first concerns the 2022 paper on MSA tar making (3). Kozowyk (1) claims that the paper’s conclusion for cognitive complexity was based on the condensation method being used in the MSA. This is incorrect. Schmidt et al. (3) show different methods by which *Podocarpus* tar could have been made. In line with the 2019 paper (2), they affirm that condensation is the simplest known tar making method (3). Instead, Schmidt et al.’s (3) conclusions are based on their finding that *Podocarpus* tar must be *manufactured*—a hitherto-unknown fact—while other natural substances with similar properties could simply have been collected. Thus, Schmidt et al. (3) do not “… showcase the inherent complexity of producing tar…” (1) rather they show that *Podocarpus* tar documents innovative behavior.
2) The second, more problematic, misconception concerns the 2019 paper on birch tar (2). The goal of this study was to establish a minimal baseline level of the difficulty of birch tar making. In 2019, the prevailing doctrine was that tar could only be produced in low-oxygen environments. This led to the view that tar making would *necessarily* require “cognitively opaque” (4) underground techniques, implying high planning depths and cumulative cultural transmission of techniques (5). However, tar also forms aboveground (also see ref. 6) even by accident. Schmid et al. (2) therefore tackled a long-known problem in Stone Age archaeology: that interpretations are overfocused on objects, bypassing investigations of how objects were produced. It is explicitly stated in ref. 2 that their findings do not mean “that [Neanderthals] were not capable of abstract thinking or high planning depths.” Thus, Kozowyk’s (1) “alternative” interpretation of the 2019 data is not, in fact, an alternative view at all.

## Conclusion

Kozowyk’s (1) claim that Schmidt et al. (3) go against Schmidt et al.’s (2) interpretation is incorrect. Schmidt et al. (3) pinpoint innovative behavior in the African MSA because *Podocarpus* tar was produced. We do believe that birch tar has the same implications for understanding human behaviors as *Podocarpus* tar. This would indeed be strengthened by comparable data on alternative adhesives from Europe (yet, contrary to what is claimed in ref. 1, only data on highly transformed industrial colophony are available from refs. 7 and 8; we need data on natural “pine resin” and other substances). Regardless, what this debate illustrates is that equating “complexity,” as in stepwise process complexity and “complex” cognition/behavior (a loosely defined qualifier, at least in archaeology), may lead to misinterpretations.
